# Chitinase genes in olive tree (*Olea europaea* L.): evolutionary dynamics and stress–responsive regulation

**DOI:** 10.3389/fpls.2026.1771535

**Published:** 2026-04-17

**Authors:** Catarina Campos, André Albuquerque, Maria do Rosário Félix, Hélia Cardoso, Dariusz Grzebelus, Lénia Rodrigues, Mariana Patanita, Joana Amaro Ribeiro, Augusto Peixe, Maria Doroteia Campos

**Affiliations:** 1 MED Mediterranean Institute for Agriculture, Environment and Development & CHANGE Global Change and Sustainability Institute, Institute for Advanced Studies and Research, Universidade de Évora, Évora, Portugal; 2 MED Mediterranean Institute for Agriculture, Environment and Development & CHANGE Global Change and Sustainability Institute, Departamento de Fitotecnia, Escola de Ciências e Tecnologia, Universidade de Évora, Évora, Portugal; 3 MED Mediterranean Institute for Agriculture, Environment and Development & CHANGE Global Change and Sustainability Institute, Departamento de Biologia, Escola de Ciências e Tecnologia, Universidade de Évora, Évora, Portugal; 4 Department of Plant Biology and Biotechnology, Faculty of Biotechnology and Horticulture, University of Agriculture in Krakow, Krakow, Poland

**Keywords:** chitinases, *Olea europaea* L., gene family, fungal infection response, regulatory elements

## Abstract

**Background:**

Chitinases (EC 3.2.1.14) are glycosyl hydrolases (GH) that break down glycosidic bonds in chitin. Plant chitinases are known to be implicated in responses to biotic and abiotic stresses, and particularly to defense against fungal pathogens by degrading fungal chitin. Olive tree (*Olea europaea* subsp. *europaea*) is a crucial fruit tree in Mediterranean ecosystems, but several pathogenic fungi can significantly affect production. The study of chitinase genes (*OeChi*) in olive tree can contribute to develop new strategies for a sustainable disease management.

**Results:**

A total of 28 chitinase genes and 5 chitinase–like genes (belonging to narbonin and SI–CPL domains) were found in the olive tree genome. Phylogenetic analysis clustered *OeChi* in two families (GH18 and GH19) and further into 5 classes, each one with its specificities regarding motifs and functional domains. It was observed that exon–intron structure was somewhat conserved in each group. Segmental and tandem duplication events were found for several GH18 genes, most of which appear to be under purifying selection. Expression profiling of *OeChi* genes under fungal infection revealed considerable variation among gene family members, with several genes showing strong induction. Promoter analysis identified a wide range of *cis*–regulatory elements associated with stress responses and hormone signaling pathways.

**Conclusions:**

Collectively, our study improved knowledge on the olive tree chitinase gene family, thus providing a valuable foundation for future strategies aimed at improving fungal disease resistance in olive tree.

## 1 Introduction

Olive tree (*Olea europaea* L. subsp. *europaea*) is one of the most economically important fruit trees in the Mediterranean basin and a vital component of the Mediterranean ecosystems, achieving in some countries a leading position in the agricultural sector (Loumou and Giourga, 2003; Sales et al., 2020). The olive tree is mainly grown to produce fruit for processing into olive oil, but also for consumption as table olives. Although the Mediterranean region remains the main growing area, this crop has spread to southern Africa, South and North America, Australia, Japan and China (FAOSTAT, accessed 01/02/2025; The Olive Tree – International Olive Council (internationaloliveoil.org), accessed 01/02/2025). In Europe the production share of olives in 2023 was 64,8% (FAOSTAT, accessed in 01/02/2025) and olive oil 72,2% (FAOSTAT, accessed in 01/02/2025). This crop is considered highly adapted and suitable to the Mediterranean–type climate (Orlandi et al., 2013; Rodrigues et al., 2024), characterized by warm dry summers and rainy winters. However, climate changes are expected to impact olive tree production in the Mediterranean Basin in future decades, and in regions of Europe such as southern Iberia the viability of olive orchards may be particularly reduced (Fraga et al., 2020).

Olive tree is known to be fairly resistant to abiotic stresses, as it can tolerate poor and dry soils, is generally drought–resistant and moderately tolerant to salinity, although genotype–related variations do exist (Fernández and Moreno, 2000; Bacelar et al., 2004). Regarding biotic stresses, soil– born, foliar and fruit fungal pathogens are responsible for economically important diseases in most olive–growing areas (Moral et al., 2017; Fernández-Escobar, 2019; Markakis et al., 2021). The pathogens can infect multiple olive organs, including leaves, flowers, fruits, roots, and stems. Many older olive orchards are still based on traditional growing systems, but new plantations are now built on more intensive growing methods. The use of new cultivars, different cultural practices and new environments can influence the effects of abiotic and biotic stresses situations (Moral et al., 2017; Fernández-Escobar, 2019). The high–density orchards may in fact contribute to increase the incidence and severity of some diseases, particularly in cultivars less suited to these systems (Landi et al., 2024). Olive anthracnose, the most significant and widespread disease affecting olive drupes, is caused by several species of *Colletotrichum* fungi (Moral et al., 2017; Materatski et al., 2018). In addition to causing premature fruit drop, these pathogens produce phytotoxins in the rotting fruit, leading to dieback in shoots and branches. Several other fungal pathogens can cause wilting, cankers, dieback, and other decline–related symptoms in olive trees (Agustí-Brisach et al., 2021). These symptoms typically manifest when the plant’s water and nutrient demands exceed the vascular tissue’s capacity to supply them. Verticillium wilt of olive, caused by the soil–borne fungus *Verticillium dahliae* Kleb., is also among the most significant constraints on olive cultivation. It leads to high tree mortality, reduced productivity and decreased fruit yield (Montes-Osuna and Mercado-Blanco, 2020). Olive fungal diseases are still primarily treated with fungicides (Moral et al., 2018; Castro et al., 2020), but EU directives for commercialization withdrew several chemical substances used for disease control since new rules were established to reduce agricultural greenhouse gas emissions in the 2015 Paris Agreement of the United Nations Framework Convention on Climate Change (Campos and Félix, 2023). In this context, understanding and enhancing the olive tree’s resistance mechanisms is critical for its sustainable cultivation. The identification of candidate genes and the development of disease–resistant cultivars applying new biotechnology techniques can be an effective and sustainable approach to control fungal diseases (Campos et al., 2021). For instance, transcriptomic changes in olive cultivars after *V*. *dahliae* inoculation have already been evaluated (Cardoso et al., 2022), with some studies showing differences in gene expression between tolerant *vs* susceptible cultivars (Gharbi et al., 2017; Leyva-Pérez et al., 2018). Amongst others, it was found an up–regulation of some chitinase genes in response to fungal infection.

Plants do not produce chitin as part of their natural metabolism or cellular structures. However, plants interact with chitin in various ways, primarily because chitin is a key component of fungal cell walls and certain insect exoskeleton. Chitinases (EC 3.2.1.14) are glycosyl hydrolases (GH) that breakdown glycosidic bonds in chitin (Grover, 2012). Chitinases are widely distributed across the plant kingdom and usually form multigene families (Tobias et al., 2017; Cao and Tan, 2019; Zheng et al., 2020; Peery et al., 2021; Haxim et al., 2022; Zhang et al., 2022; He et al., 2023). Plant chitinases have been shown to play a crucial role in responding to both biotic and abiotic stresses (Vaghela et al., 2022), being nowadays promising tools for crop improvement.

Plant chitinases are grouped into glycoside hydrolase families based on sequence and domain structure determining five main classes (Class I–V) (Passarinho and de Vries, 2002). The family GH18 includes chitinases of classes III and V, whereas chitinases I, II and IV are part of the family GH19 (Grover, 2012). Class I chitinases have a highly–conserved N–terminal cysteine–rich region and a chitin–binding region (CBD), a highly variable proline–rich hinge, a catalytic domain and usually a C–terminal extension that facilitates their targeting to vacuolar and intracellular transport (Graham and Sticklen, 1994). Class II lacks an N–terminal cysteine–rich region and a CBD, but its catalytic region is highly similar to the amino acid sequence of class I chitinases (Graham and Sticklen, 1994). They are generally acidic and secreted to the extracellular space and often induced by pathogen infection (Patil et al., 2000; Grover, 2012). Class IV chitinases have a chitin–binding region and a catalytic domain but generally miss a C–terminal extension. However, deletions or the absence of these regions shorten the final protein product relative to class I chitinases (Graham and Sticklen, 1994; Tobias et al., 2017; Tobias et al., 2017). Class III chitinases lack a chitin–binding region and have little sequence identity to GH19 chitinases (Graham and Sticklen, 1994; Grover, 2012). The GH18 class V members are more similar to fungal and bacterial chitinase than other plant chitinases (Melchers et al., 1994), and both classes III and V present lysozyme activity and chitinase activity (van Loon et al., 2006). Narbonin and SI–CLP (stabilin–1 interacting chitinase–like protein), which belong to the chitinase–like proteins (CPLs), have been found in several plant species and are related to the GH18 family, but lack a binding/catalytic activity due to the presence of substitutions in the chitin-binding domain. CPLs however, have been reported to participate in plant growth and in several developmental processes, as well as responses to stresses (Tyler et al., 2010; Kesari et al., 2015).

Compared to annual herbaceous plants, perennial woody plants have significantly longer generation times. Throughout their life cycles, they are constantly challenged with a wide range of changing abiotic and biotic stresses. One way that longer–lived species have compensated for the lifetime of biotic stresses, especially from fungal pathogens, is by expanding the repertoire of chitinase genes within their genomes. In the study here presented, the chitinase and CPL genes in *O. europaea* subsp. *europaea* (*OeChi*) were investigated, focusing on their phylogenetic relationships, gene structures, and gene duplication events. Additionally, the expression patterns of *OeChi* and CPL genes were analyzed in response to fungal infections. This work aims to contribute to the development of effective genetic tools for improvement of olive tree resistance to fungal infections.

## 2 Materials and methods

### 2.1 Identification of the chitinase gene family members in *Olea europaea* subsp. *europaea* (*OeChi*)

The genome and gene annotation files of *O. europaea* subsp. *europaea* cv. “Farga” (Genome assembly OLEA9) were retrieved from *Ensembl Plants* (release 59), and chitinase genes were identified through BlastX against Viridiplantae protein sequences, using the software OmicsBox 3.3.2. (BioBam Informatics, Valencia, Spain). The key parameters were set as default, and the cutoff value was set as 1E–3.

Candidate chitinase proteins were further validated by confirming the presence of conserved GH18 or GH19 domains using Pfam (https://www.ebi.ac.uk/interpro/entry/pfam), InterProScan (Jones et al., 2014), and HMMER (https://www.ebi.ac.uk/Tools/hmmer/search/hmmsearch). Only sequences with complete conserved domains were retained. Proteins lacking essential catalytic residues were classified as chitinase-like. No transcriptomic filtering was applied, as the identification was based on genome annotation.

### 2.2 Phylogenetic relationships, gene organization, and conserved motif analysis

Sequences of chitinase proteins from *O. europaea* subsp. *europaea* var. *sylvestris*, *Arabidopsis thaliana* (Ensembl Plants, TAIR10), *Sesamum indicum* (Ensembl Plants, S_indicum_v1.0), *Vitis vinifera* (Ensembl Plants, ASM3070453v1), *Manihot esculenta* (Ensembl Plants, M.esculenta_v8), *Medicago truncatula* (Ensembl Plants, MtrunA17r5.0_ANR), *Populus trichocarpa*, *Malus domestica* Golden (Ensembl Plants, ASM211411v1) and *Mimulus guttatus* (https://phytozome–next.jgi.doe.gov/info/Mguttatus_v2_0) were obtained by BlastP searches using *O. europaea* subsp. *europaea* chitinases protein sequences as a query. NCBI Conserved Domain Database (CDD) database was used to confirm all the sequences.

The full–length amino acid sequences of chitinase proteins from the above species were used to create multiple alignments using MUSCLE (Edgar, 2004) with default parameter sets. A maximum likelihood (ML) phylogenetic tree (1000 bootstrap replicates) was built using the CLC Genomics Workbench v 11.0.1. (Qiagen, Aarhus).

The exon–intron organization of *OeChi* was visualized using TBtools–II (Chen et al., 2023), according to their genome annotation files. Motifs and domains were visualized using TBtools, using the output of MEME Suite (maximum –20 conserved motifs and a motif width of 10 to 50 amino acids) (Bailey et al., 2006) and CDD database at NCBI (Wang et al., 2023) (expect value threshold = 0.01, composition–corrected scoring), respectively.

### 2.3 Chromosomal location and gene duplication analysis

Synteny relationships of chitinase genes between *O. europaea* subsp. *europaea, O. europaea* subsp. *europaea* var. *sylvestris* and *Sesamum indicum* (order Lamiales, same as *O. europaea*) were visualized using the Multiple Synteny Plot in TBtools. Duplicated chitinase gene pairs in *O. europaea* were identified using TBtools by conducting an all–versus–all BLASTP search (E–value ≤ 1e−5) followed by MCScanX collinearity analysis (Wang et al., 2012) with genome annotation files. Segmental duplications were defined based on syntenic blocks, and tandem duplications as adjacent homologous genes separated by 5 or fewer genes within a 100 kb region on the same chromosome (Zhao et al., 2019). Nonsynonymous (Ka) and synonymous (Ks) substitution rates and Ka/Ks ratios for both tandem and segmental pairs were calculated in TBtools using the Ka/Ks Calculator module, and duplication relationships were visualized with the Advanced Circos function.

### 2.4 Physicochemical characterization of OeChi proteins and prediction of subcellular location

The gene ID, chromosomal location, coding sequence and putative amino acid sequence length of each *OeChi* were retrieved from the EnsemblPlants database. The predicted physicochemical properties of *OeChi*–encoded peptides were estimated using The ProtParam tool of ExPASy Server (https://web.expasy.org/protparam/, accessed on 20 February 2025), including protein molecular weight (MW) and isoelectric point (pI) values. The Plant–mPLoc Server (http://www.csbio.sjtu.edu.cn/bioinf/plant/) was used for the prediction of subcellular location of proteins. The SignalP 6.0 online server (Teufel et al., 2022) was used to identify signal peptides in OeChi proteins.

### 2.5 *In silico* identification of regulatory elements within *OeChi* genic regions

To predict potential *cis*–acting elements in *OeChi* gene sequences the promoter sequence (up to the 2.0 kb DNA sequence upstream of the start codon) of each *OeChi* was retrieved from the *O. europaea* subsp. *europaea* genome (Genome assembly OLEA9 in Ensembl Plants). The *cis*–acting elements in each gene were predicted using the PlantCARE online tools (Lescot et al., 2002).

To search for the presence of long terminal repeats (LTRs) at the OE9A061598 large introns (intron 1 with 5550 bp and intron 2 with 4430 bp), the DNA sequences were self–aligned using Blast2Seq tool at NCBI. The region flanked by LTRs was used as a query to search for related elements in RepBase using Censor (Kohany et al., 2006) and blastn at NCBI restricted to *Olea europaea* (taxid: 4146; accessed on 21 October, 2024). Additional manipulations and alignments were performed in BioEdit 7.2.5 (Hall, 1999).

### 2.6 Expression analysis of *OeChi* genes

#### 2.6.1 *In silico* analysis of *OeChi* expression in different tissues/organs

The OliveAtlas database (https://www.oliveatlas.uma.es/easy_gdb/index.php) (Jiménez-Ruiz et al., 2020) was used to retrieve RNA–seq data of *OeChi* from *O. europaea* subsp. *europaea* cv. ‘Picual’ in different tissues/organs from 10–year–old olive trees growing under field conditions (roots, stems, meristems, leaves, flowers at anthesis and fruits) (Ramírez-Tejero et al., 2020), from 4 months old trees (roots and leaves) (Leyva-Pérez et al., 2015), from mature pollen and pollen 6 hours after germination (Bullones et al., 2023), and from mature seed. Chitinase and CPL genes in OliveAtlas were identified by Blast analysis of the previously identified genes. A heat map using log transformed transcripts per million (TPM) for each gene was generated by TBtools software.

#### 2.6.2 *In silico* analysis of *OeChi* expression in response to infection with *Verticillium dahliae*

The OliveAtlas database was used to retrieve RNA–seq data of chitinase genes from *O. europaea* subsp. *europaea* cv. ‘Picual’ in response to infection with the soil–borne fungus *Verticillium dahliae* (Leyva-Pérez et al., 2015), the causal agent of Verticillium wilt of olive. Expression data was from leaves infected with *V. dahliae* for 15 days and respective control, and for roots infected with *V. dahliae* for 48 hours, 7 days and 15 days, and respective control. A heat map using log transformed TPM for each chitinase gene was generated by TBtools software.

#### 2.6.3 Analysis of *OeChi* expression in response to *Colletotrichum nymphaeae* inoculation through RT–qPCR

Thirteen olive plants cv. ‘Galega vulgar’ obtained from *in vitro* culture (Peixe et al., 2007), were used in the experiment. The plants were transplanted into trays with commercial substrate (Plantobalt, Estonia) and kept in a greenhouse with a mean temperature of 26 °C for 90 days. Following this, plants were transplanted into pots and kept in a room under controlled temperature (22–25 °C), with a 14 hours’ photoperiod.

An isolate of the fungus *Colletotrichum nymphaeae* belonging to the collection of the Mycology Laboratory, Mediterranean Institute for Agriculture, Environment and Development (MED), University of Évora, Portugal, was selected for the experiment. The fungus was cultured on potato dextrose agar (PDA) for 28 days at 24 °C under a 12 hours’ photoperiod. Olive plants were inoculated by spraying with *C. nymphaeae* suspensions containing 10^6^ conidia mL^−1^ as previously described (Gomes et al., 2009). After inoculation, plants were covered with plastic bags for 48 hours to conserve moisture in the leaves. Samples consisted of 5–6 leaves taken before inoculation (T0), 10 days after the inoculation (T1) and –30 days after inoculation (T2). Samples were macerated in liquid nitrogen and kept at –80 °C until further use.

To confirm olive infection after fungi inoculation, gDNA was extracted from the 13 olive plants at T0 and at T2 and from *C. nymphaeae* fungus structures (used as qPCR positive control) using the ‘DNeasy Plant mini kit’ (Qiagen, Hilden, Germany), following the manufacturer’s protocol. Fungi detection and quantification in plants was performed by qPCR following the procedure described by Ribeiro et al. (2022) (Ribeiro et al., 2022) using the Lime_1F and Lime_2R primers (Lime_1F: 5’–GCCAACAAATAAACGCCACT–3’, Lime_2R: 5’–GACTTATTCGGTGACGTGCC– 3’) (**Supplementary Figure 1**) (Azevedo-Nogueira et al., 2021). This step was crucial for the selection of plants for expression analysis.

For expression analysis, total RNA extraction was carried out on five infected plants in three time points (T0, T1 and T2) using the ‘RNeasy Plant mini kit’ (Qiagen, Hilden, Germany). The purity of each RNA sample was assessed through absorbance ratio analysis (A260/280 and A260/230), and integrity was evaluated by electrophoresis on an agarose gel run under denaturing conditions. After purity and quality control, total RNA (1 μg) was reverse transcribed with the Maxima First Strand cDNA Synthesis Kit (Thermo Scientific, Waltham, MA, USA). The selected reference genes were *Actin* (*ACT*), *Glyceraldehyde–3–phosphate dehydrogenase* (*GAPDH*) and *Elongation facto*r (*EF1a*) (Ray and Johnson, 2014; Velada et al., 2018). RT–qPCR was performed using SYBRGreen chemistry on a LineGene9600Plus (BIOER, Hangzhou, China), following the procedure and conditions previously described (Monteiro et al., 2024). Specificity of primers was confirmed by melting curve analysis (**Supplementary Figure 2**).Stability of reference genes and normalization factors were obtained with the software *geNorm* (Vandesompele et al., 2002). The target *OeChi* genes, belonging to different classes from the GH18 and GH19 families, are listed in **Supplementary Table 1**. Primers were designed using the Primer 3.0 software using the default parameters (https://www.primer3plus.com/index.html).

To investigate target gene expression, Ct values were regressed against the log of the produced cDNA standard curve. The value of normalized arbitrary units of the target genes was then determined for each sample using the reference genes normalization parameters. The gene expression values were transformed by using a log_e_ transformation to ensure a normal distribution and homogeneity of variance, using the software IBM SPSS Statistics (IBM SPSS Statistics for Windows, version 21.0, Armonk, NY IMB Corp), and considered a significant *p*–value < 0,05. Multiple comparisons amongst time points were performed through ANOVA using the Bonferroni post–hoc test (Jafari and Azuaje, 2006).

## 3 Results

### 3.1 Genome–wide identification and expansion patterns of *OeChi* genes in *O. europaea* subsp. *europaea*

The genome and reference transcriptome sequences of *O. europaea* subsp. *europaea* were used for genome–wide exploration and phylogenetic analysis of the chitinase gene family. Databases including HMMER, PFAM, InterProScan and CDD were used to confirm and visualize olive tree chitinases’ domains. We accurately identified –28 putative canonical chitinase genes and five chitinase–like genes in *O. europaea* (**Table 1**). Eighteen chitinase genes are members of the GH18 family and 10 genes belong to the GH19 family. The chitinase–like genes belong to the narbonin and SI–CLP lineages, which lack the conserved catalytic residues required for chitin hydrolysis (Kesari et al., 2015) and are more related to the GH18 family. Of the total of the 33 genes, 17 could be located in the *O. europaea* chromosomes and the others are specified in their contig (**Supplementary Table 2**).

**Table 1:** Details of chitinase and chitinase–like genes identified in O. europaea subsp. europaea.

Gene ID	Chr.	Class	Predicted location plant – mPLoc	Signal peptide probability
OE9A060505		GH19 Class I	Vacuole	0.9644
OE9A040457	18	GH19 Class I	Vacuole	0.5774
OE9A106107		GH19 Class I	Cell wall	0.9656
OE9A109397		GH19 Class IV	Cell wall	NA
OE9A112876		GH19 Class II	Extracell.	0.9745
OE9A087887	4	GH19 Class II	Vacuole	0.9806
OE9A025476		GH19 Class II	Vacuole	0.9804
OE9A068866		GH19 Class II	Vacuole	0.7526
OE9A017959	4	GH19 Class II	Vacuole	0.7927
OE9A081614	4	GH19 Class II	Vacuole	0.5448
OE9A061598	6	GH18 Class III	Extracell.	NA
OE9A015656	6	GH18 Class III	Vacuole	0.9658
OE9D001465	7	GH18 Class III	Cell wall	NA
OE9A019142	7	GH18 Class III	Cell wall	0.9736
OE9A070421	7	GH18 Class III	Vacuole	0.9548
OE9A116561	11	GH18 Class III	Vacuole	NA
OE9A048822		GH18 Class III	Vacuole	0.9700
OE9A113003		GH18 Class III	Vacuole	0.9794
OE9A107410		GH18 Class III	Vacuole	0.9778
OE9A116045		GH18 Class III	Vacuole	0.9750
OE9A010255		GH18 Class III	Vacuole	0.9749
OE9A053601	12	GH18 Class III	Vacuole	0.9690
OE9A032682	12	GH18 Class III	Vacuole	0.9731
OE9A111013		GH18 Class III	Vacuole	0.9731
OE9A032348		GH18 Class V	Cell wall	NA
OE9A012697		GH18 Class V	Cell wall	0.9794
OE9A070518		GH18 Class V	Cell wall	0.6086
OE9A027465	21	GH18 Class V	Cell wall	0.6602
OE9A105678	9	Chitinase–like GH18 narbonin	Cell wall	0.9799
OE9A048255	9	Chitinase–like GH18 narbonin	Cytoplasm	NA
OE9A016370		Chitinase–like GH18 narbonin	Cell membrane; Cell wall	0.9280
OE9A066109	1	Chitinase–like GH18 narbonin	Cell wall	NA
OE9A118797	7	Chitinase–like GH18 SI–CLP	Chloroplast	NA

The length of the 33 predicted proteins ranged from 125 (OE9D001465) to 434 (OE9A118797) amino acid residues. The predicted molecular weight (MW) and isoelectric point (PI) ranged from 13319.37 Da (OE9D001465) to 49656.73 Da (OE9A118797) and from 4.27 (OE9A111013) to 9.42 (OE9A032348), respectively (**Supplementary Table 2**). A signal peptide located at the N–terminus of most plant chitinases is responsible for their secretion after posttranslational modification (Taira et al., 2009). As shown in **Table 1**, 25 of the 33 proteins (75.8%) possess N–terminal signal peptides, which suggests that these proteins have potential travel functions. Two chitinase–like proteins (CLP) from the narbonin groups as well as SI–CLP do not possess signal peptide, and also one GH19 class IV, three GH18 class III and one GH18 class V proteins (**Table 1**). The majority of GH19 members are predicted to be localized in the vacuole, as well as most of GH18 class III chitinases. CLP narbonins and class V are mostly predicted to be located in cell wall, and GH18 SI–CLP in the chloroplast (**Table 1**).

**Figure 1:**
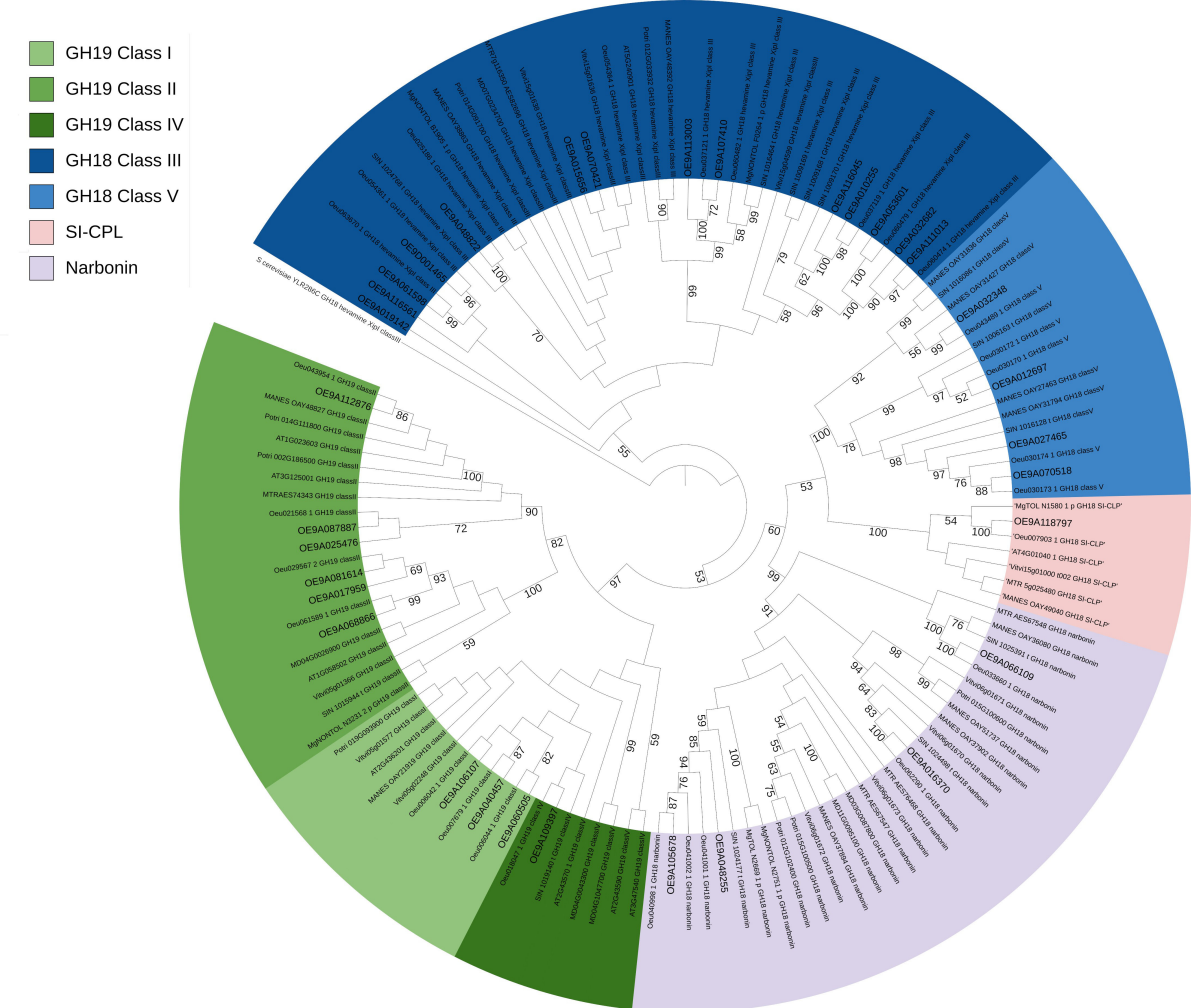
Phylogenetic tree of chitinase proteins in O. europaea subsp. Europaea (OE9A), O. europaea subsp. europaea var. sylvestris (Oeu), S. indicum (SIN), A. thaliana (AT), V. vinifera (Vitvi), *M. esculenta* (MANES), M. truncatula (MTR), *P. trichocarpa* (Potri), *M. guttatus* (Mg) and *Malus domestica* Golden (MD). The five classes (I – V) plus narbonin and SI–CPL were assigned according to the ortholog structure and function.

The GH18 subfamily was branched into two classes (hevamine XipI Class III and Class V) whereas the GH19 was grouped in the three classes (class I (lysozyme family protein), class II and class IV) (**Figure 1**). CPLs from the narbonin and SI–CLP (homolog of the stabilin–1 interacting chitinase–like protein) groups were clustered more closely to the GH18 class V (**Figure 1**). As expected, *OeChi* sequences from *O. europaea* subsp. *europaea* closely clustered with *O. europaea* subsp. *europaea* var. *sylvestris* (**Figure 1**).

To explore the evolutionary relationships within the *OeChi* gene family, a maximum likelihood (ML) phylogenetic tree was constructed using chitinase protein sequences from *Olea europaea* and representative plant species. The selected species encompassed (i) intraspecific *Olea* diversity, (ii) closely related Lamiales taxa, and (iii) phylogenetically distant but well-annotated core eudicots. The phylogenetic analysis clearly distinguished OeChi members into two major groups corresponding to the GH18 and GH19 glycoside hydrolase families (**Figure 1**). These groups were further subdivided into five classes (Classes I–V), consistent with the widely accepted classification of plant chitinases reported in species such as *A. thaliana* (Passarinho and de Vries, 2002), *M. domestica* (Haxim et al., 2022), and *Populus trichocarpa* (Zhang et al., 2022). Specifically, the GH18 subfamily was divided into Class III (hevamine/XipI type) and Class V, whereas the GH19 subfamily comprised Class I (lysozyme-like proteins), Class II, and Class IV (**Figure 1**). This classification is in agreement with previously established chitinase grouping systems based on sequence homology and domain architecture. Chitinase-like proteins (CPLs), including members of the narbonin and SI-CLP (stabilin-1 interacting chitinase-like protein homolog) groups, clustered more closely with GH18 Class V, supporting their evolutionary relationship with catalytically active chitinases despite lacking key enzymatic residues (**Figure 1**). As expected, OeChi sequences from *Olea europaea* subsp. *europaea* showed the closest phylogenetic relationship with those from *Olea europaea* subsp. *europaea* var. *sylvestris*, reflecting their high genetic similarity (**Figure 1**).

**Figure 2:**
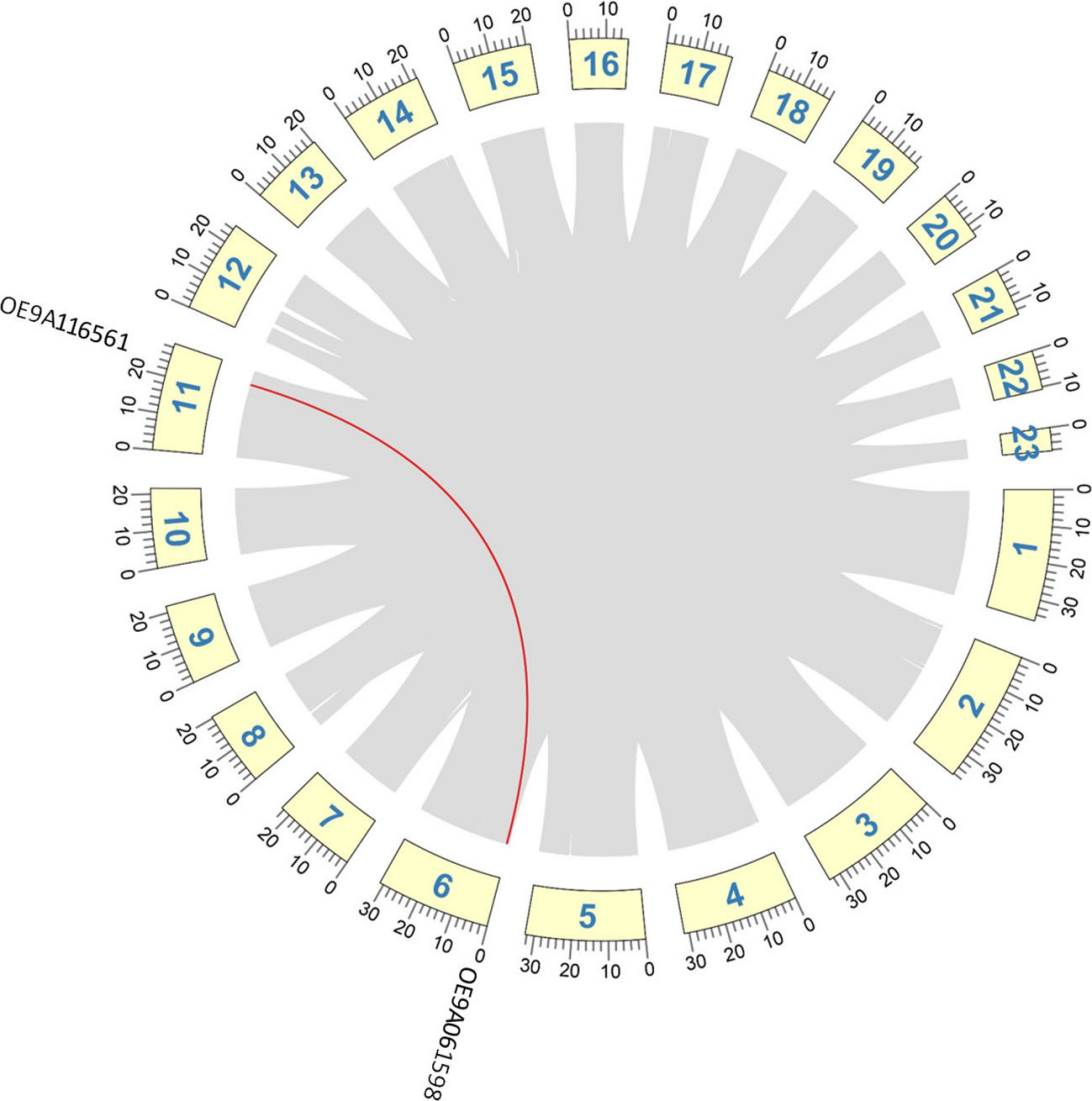
Collinearity analysis of chitinase genes in *O. europaea*, showing the distribution of segmental duplication across chromosomes. Chromosomes are represented by yellow boxes, and the chromosome numbers are indicated at the inside of the box. Segmental duplicated genes are connected with red lines. The grey lines indicate all syntenic blocks within the genome.

MCScanX tool was employed to identify duplication events in *O. europaea* subsp. *europaea* chitinases and CLPs. Segmentally duplicated genes were identified by collinearity analysis. One pair of the GH18 Class III genes was segmentally duplicated between chromosomes 6 (OE9A061598) and 11 (OE9A116561) (**Figure 2**), and three gene pairs were identified as tandemly duplicated: the GH18 Class III OE9A070421 and OE9A019142 in chromosome 7, the GH18 Class III OE9A032682 and OE9A053601 in chromosome 12 and the narbonins OE9A105678 and OE9A048255 in chromosome 9. To further explore the evolutionary history of the chitinase family, we calculated the *K*_a_ and *K*_s_ substitution rates for duplicated chitinase gene pairs. Tandem GH18 Class III and narbonin were subjected to purifying selection (*K*_a_/*K*_s_ < 1), but for the segmental duplicated pair a great sequence divergence was observed, therefore there was a synonymous substitution saturation and *K*_s_ could not be reliably estimated (**Supplementary Table 3**).

**Figure 3:**
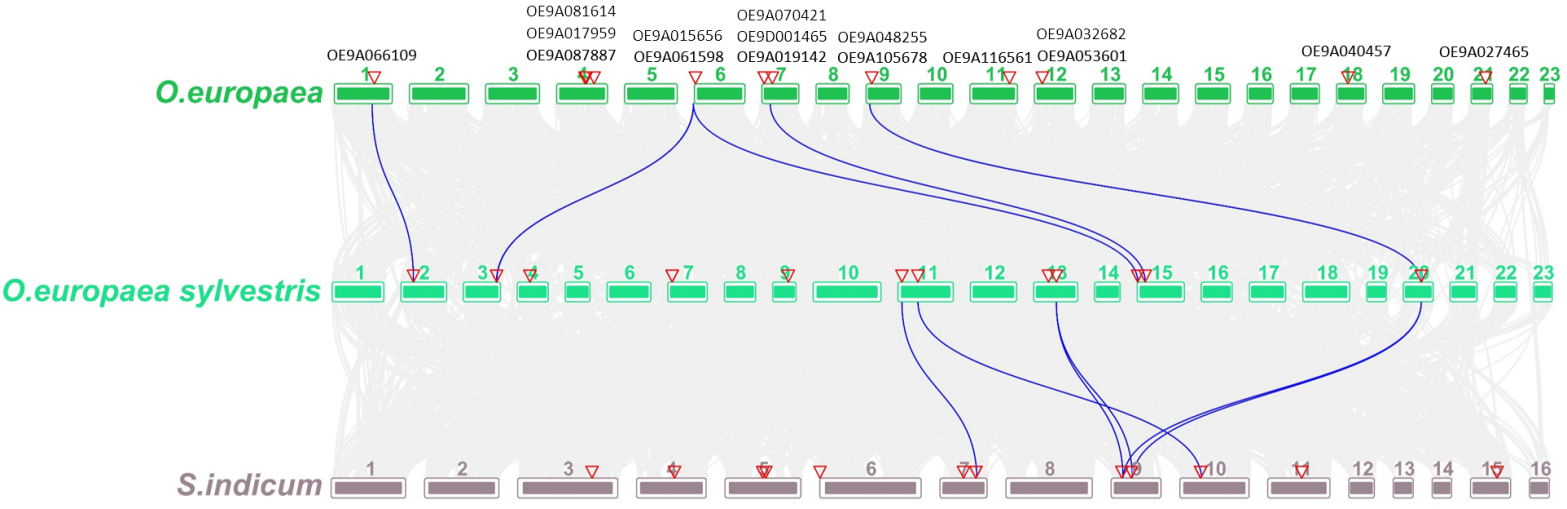
The homology analysis of chitinases from *O. europaea subsp. europaea*, *O. europaea subsp. europaea* var. *sylvestris* and S*. indicum* genomes. The gray lines in the background indicate the homologous relationship within *O. europaea subsp. europaea* and the other two plant genomes, while the blue lines highlight the members of the chitinase gene family that have a homologous relationship between the three genomes. Numbers indicate chromosome number and triangles indicate the location of the genes.

To understand the orthologous relationships between *O. europaea* subsp. *europaea*, its wild relative *O. europaea* subsp. *europaea* var. *sylvestris* and *S. indicum*, we further analyzed the synteny of *OeChi* genes and chitinase–like genes. There were found 5 collinear gene pairs between *O. europaea* subsp. *europaea* and *O. europaea* subsp. *europaea* var. *sylvestris*, being only one shared with *S. indicum* (**Figure 3**).

### 3.2 Conservative protein motif analysis and gene structure of chitinases in *O. europaea* subsp. *europaea*

**Figure 4:**
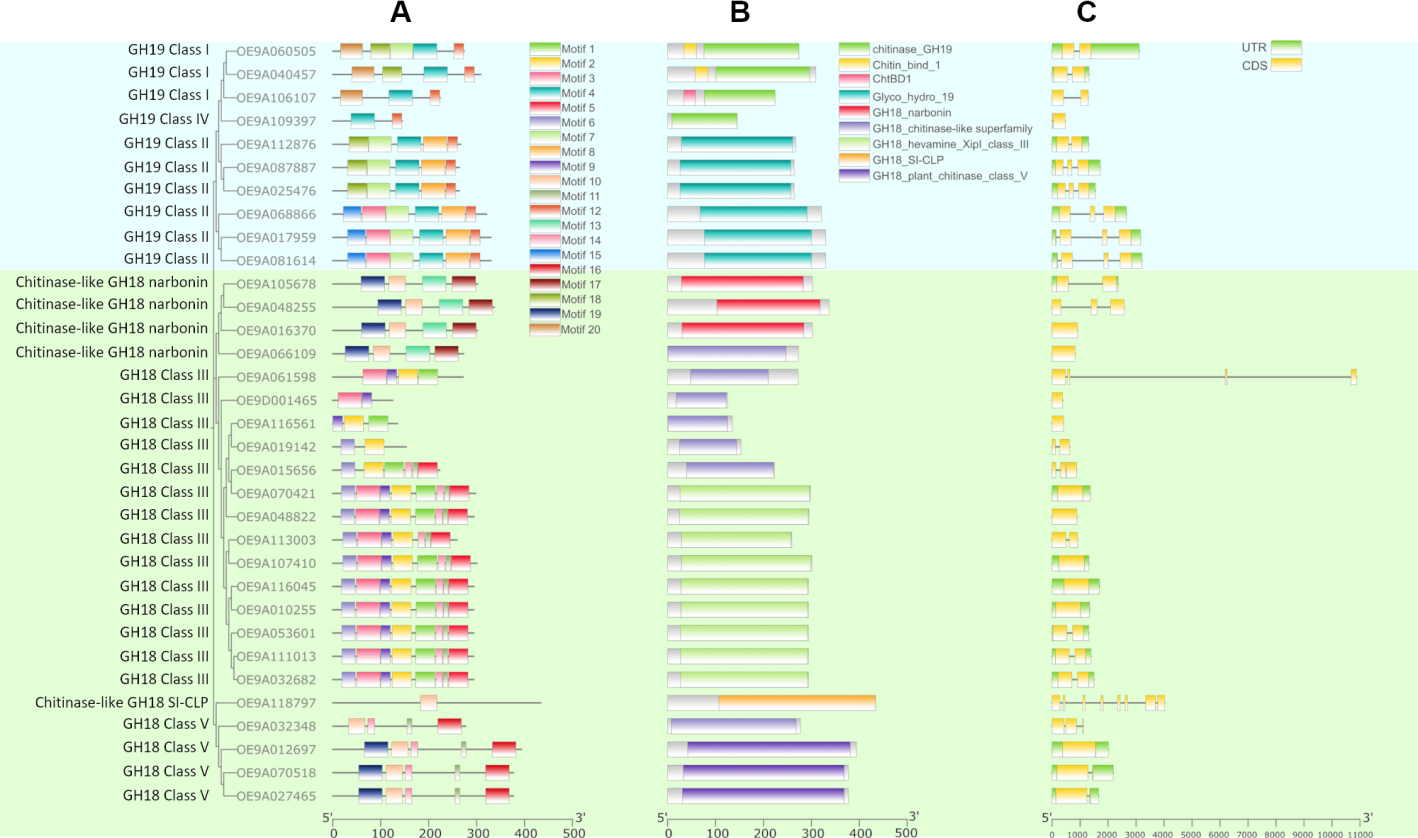
Motif composition, conserved domains and gene structure of predicted GH18 and GH19 chitinases of *O. europaea* subsp*. europaea*. **(A)** Conserved motifs of *O. europaea* chitinases were identified by MEME, and different motifs are represented by specific colors. **(B)** Catalytic domains of *O. europaea* chitinases, as identified by CDD database. **(C)** Gene structure of the 33 predicted chitinases and chitinase-like genes in olive tree. Yellow boxes and black lines represent exons and introns, respectively. Green boxes represent untranslated regions (UTRs).

Twenty conserved motifs and 9 domains were identified amongst the *O. europaea* chitinases and chitinases–like genes. All *OeChi* members of the GH19 subfamily shared motifs 4 and 12, which were absent in the other genes (**Figure 4A****;** **Supplementary Table 4**). Motif 20 was exclusive to Class I. Among the Class II members, three genes contained motif 18, whereas the remaining three harbored motif 15 (**Figure 4A**). Motifs 2, 5, 6, and 9 were detected exclusively in GH18 Class III members, while motif 16 was restricted to GH18 Class V (**Figure 4A**). The narbonin group, SI–CPL, and GH18 Class V members shared motif 10, which was not identified in any other gene. In addition, motif 17 was exclusive to narbonin CPLs, and motif 19 was restricted to Class V and narbonins (**Figure 4A**, **Supplementary Table 4**).

The chitinase_GH19 domain was only found in classes I and IV, and a CBD, which has a role in the recognition and binding of the chitin subunits was also found in GH19 class I (**Figure 4B**). On the contrary, the domain Glyco–hydro_19 was restricted to class II members. From the 14 OeChi sequences clustered with GH18 hevamine XipI class III, the domain GH18_hevamine_XipI_class_III was found only in 9 sequences. The remaining five sequences have the domain GH18_chitinase–like super family (**Figure 4B**).

Exon–intron analysis revealed some differences in gene structure, particularly on intron number, between GH18 and GH19 subfamilies, being significantly higher in GH19 (t–test, *P* < 0.05) (**Figure 4C**, **Supplementary Table 4**). All ten GH19 sequences present introns (ranging one to three), whereas of the 23 GH18 sequences, 10 members lack intron sequences, 8 have a single intron and three have two introns (**Figure 4C**). Two genes are clearly distinct from the others: the GH18 class III OE9A061598, which has two exceptionally large introns (5550 bp and 4430 bp, respectively) and a smaller one, and the SI–CLP gene, which presents 7 introns (**Figure 4C**). Most of the closely related genes show similar exon–intron structure, particularly in the GH19 subfamily and some show exon/intron size conservation (**Figure 4C**).

### 3.3 Prediction of potential cis–acting elements in *OeChi* genes from *O. europaea* subsp. *europaea*

**Figure 5:**
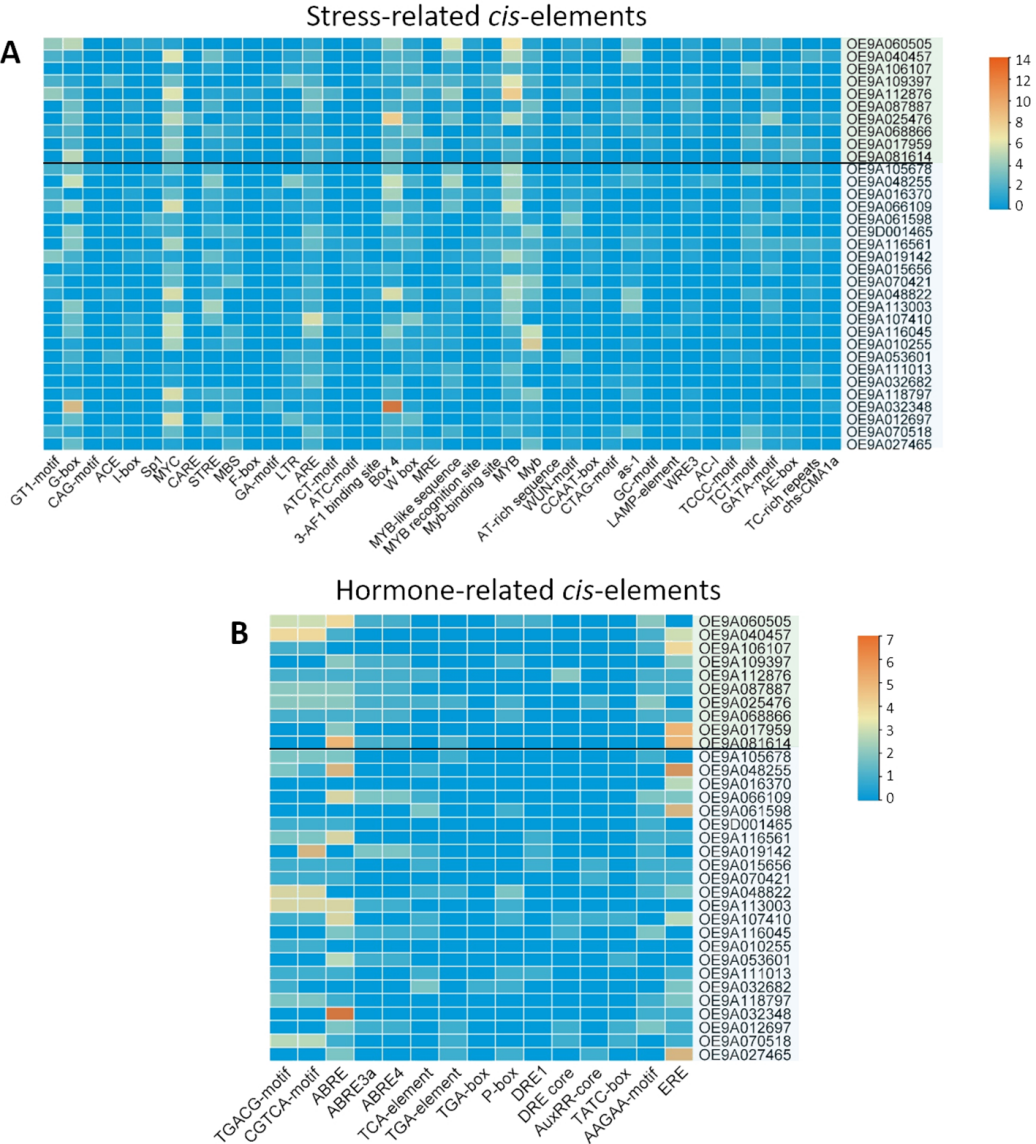
Frequency of *cis–*elements predicted in the promoter region of *OeChi* gene family members (2kb upstream of the start codon). **(A)** Stress–related *cis*–elements, **(B)** Hormone–related *cis*–elements. The color scale represents the number of *cis*–elements found in the promoter regions of *OeChi*, ranging from 0 (dark blue) to 14 (dark orange) for the stress–related elements, and 0 to 7 for the hormone–related elements.

To explore the function and regulatory mechanism of *OeChi* genes, the 2000 bp promoter region upstream of the start codon was searched for each *O. europaea* subsp. *europaea OeChi* sequence at the PlantCARE database. We found multiple *cis*–elements involved in response to various stresses and hormone–related (**Figure 5**). The most abundant stress–related elements in *OeChi* promoters were G–box and box 4 (involved in light responsiveness), MYC and MYB (involved in drought and salt induced–stress, but also abscisic acid (ABA) signaling and jasmonic acid (JA)–mediated defense responses, and ARE (Anaerobic Response Element). The genes with highest number of stress–related elements were OE9A025476 (GH19 class II), OE9A048822 (GH18 hevamine class III) and OE9A048255 (GH18 narbonin), with 47, 46 and 46 *cis*–elements, respectively (**Figure 5A**).

Hormone–responsive *cis*–elements including ABRE (ABA response), CGTCA–motif and TGACG–motif (methyl jasmonate (MeJA) response) and ERE (ethylene–responsive element) were the most abundant across *OeChi* genes (**Figure 5B**). The genes with the highest number of hormone–related elements were the GH18 narbonin OE9A048255 and the GH18 hevamine class III OE9A113003 and OE9A048822 (**Figure 5B**).

At least one *cis*–element known to be involved in biotic stress responses, such as W box (pathogen defense and systemic acquired resistance), ERE (ethylene–mediated defense pathway) and TGACG–motif was found in the majority of the 33 *OeChi* genes (**Figure 5B**). For instance, W box was found in 79% of the genes, being more abundant in OE9A107410 (GH18 hevamine class III), OE9A068866 and OE9A112876 (GH19 class II) and OE9A012697 (GH18 class V) (**Figures 5A, B**).

### 3.4 Identification and characterization of intronic LTR retrotransposon insertions

The large size of intron 1 (5550 bp) and intron 2 (4430 bp) of OE9A061598 in comparison with the remaining *OeChi* members, which range from 28 to 1120 bp, has led us to investigate in more detail the sequence composition of this gene. In the case of intron 1, a copy of an LTR–*Copia* retrotransposon spanning almost the entire intron was identified (**Supplementary Figure 3**). In Repbase it is the most similar to *Copia*–74 MN from *Morus notabilis*, and according to the data of Barghini et al. (Barghini et al., 2015) it is similar to 72239_E (KM577473) and 83850_A (KM577352) of olive tree. Regarding the intron 2 no repeats were identified.

### 3.5 Expression of *O. europaea* chitinase genes in different tissues/organs

**Figure 6:**
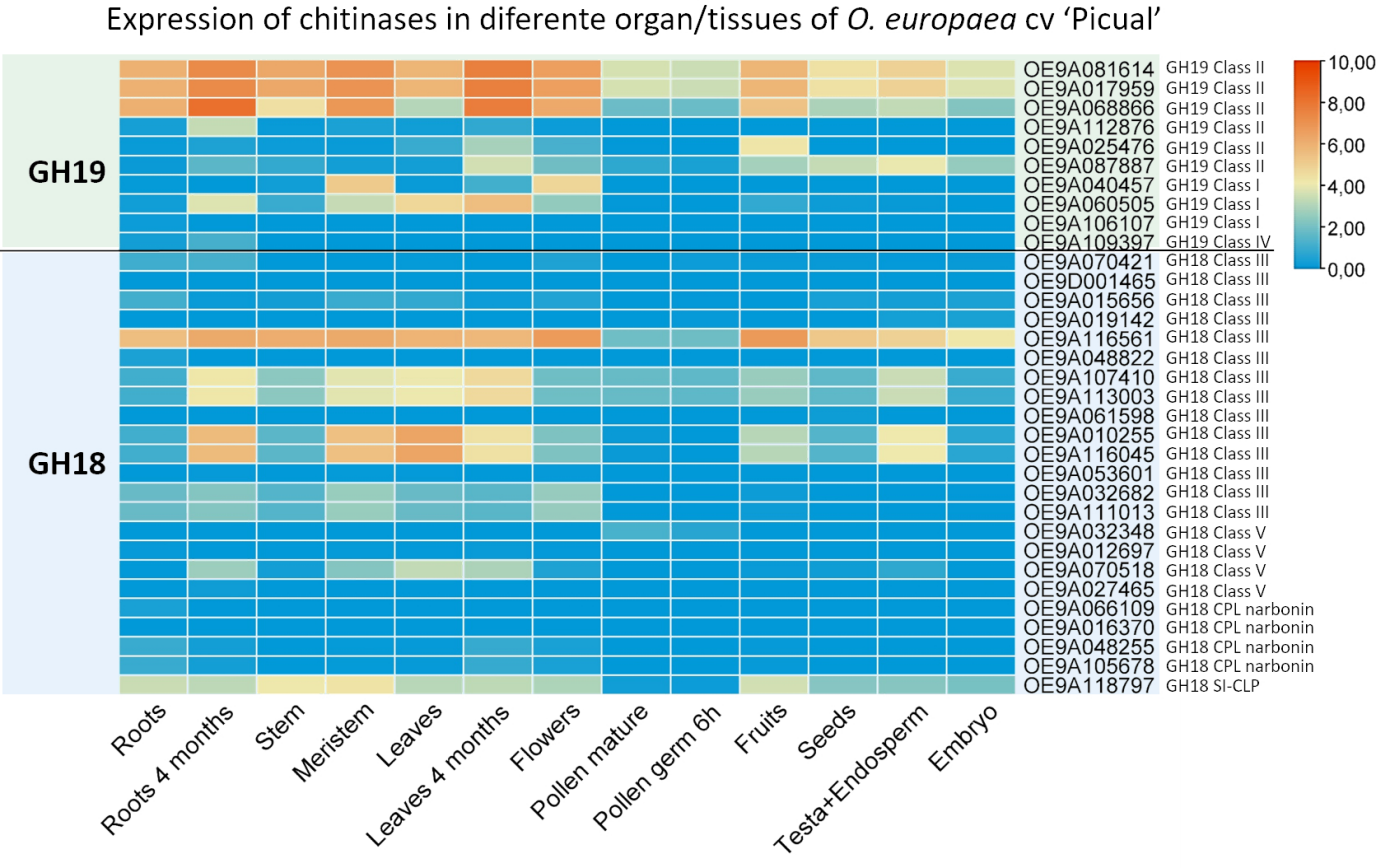
Expression profile of *OeChi* family members in different organ/tissues of *O. europaea* cv ‘Picual’, as retrieved from OliveAtlas database. The color scale represents the relative transcript levels of *OeChis* (log transformed TPM), with blue denoting low expression and red denoting high expression.

Expression patterns of *OeChi* genes in different organs/tissues were analyzed using RNA–seq data retrieved from OliveAtlas database. The findings suggest that *O. europaea* chitinases exhibit distinct roles across different organs. While most chitinase genes showed very low expression levels in all tissues, several genes such as OE9A081614, OE9A017959 and OE9A068866 (GH19 class II), OE9A116561 (GH18 hevamine class III) and OE9A118797 (GH–18 SI–CLP) displayed a ubiquitous expression across tissues, with higher transcript accumulation in roots, stem, meristem, leaves and flowers (**Figure 6**). On the other hand, the expression in pollen was always very low. A clear tissue–specific expression was observed for instance for OE9A040457 and OE9A060505 (GH19 class I), OE9A032682 and OE9A111013 (GH18 class III) and OE9A070518 (GH18 class V), with higher expression levels in roots, meristem, leaves and flowers (**Figure 6**).

### 3.6 Expression of *O. europaea* chitinase genes in response to fungal inoculation

To investigate the expression of *OeChi* genes in response to fungal infection, we made two approaches: the analysis of expression data retrieved from the OliveAtlas database, of leaves and roots of *O. europaea* cv. ‘Picual’ following inoculation with *V. dahliae* (Leyva-Pérez et al., 2015), and an experimental trial using cv. ‘Galega vulgar’ infected with *C. nymphaeae*.

**Figure 7:**
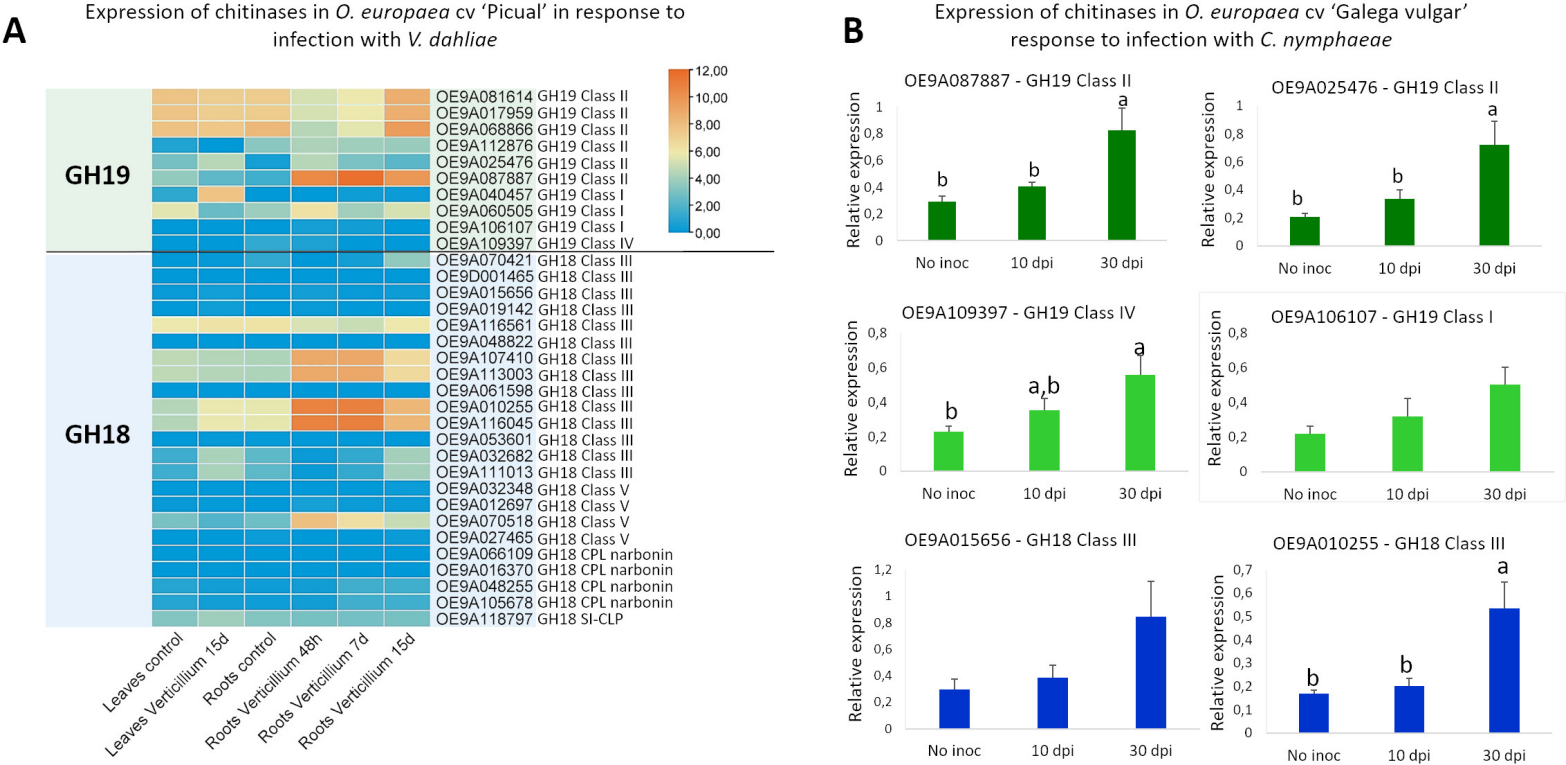
Expression profile of *OeChi* family members in response fungal infection. **(A)** Heat–map of *OeChi* expression in leaves and roots of *O. europaea* cv ‘Picual’, as retrieved from OliveAtlas database. **(B)** Expression of *OeChi* in cv ‘Galeva vulgar’ leaves infected with *C. nymphaeae*. Different letters indicate significant differences in gene expression between time points (*P* < 0.05).

Regarding the ‘Picual’ infection with *V. dahliae*, we found that several genes, from both GH18 and GH19 were up–regulated following fungus infection, mostly in the roots (**Figure 7A**). The GH19 class II members OE9A087887, OE9A081614, OE9A068866 and OE9A017959 were particularly responsive to the infection. The GH18 class III members OE9A010255, OE9A116045, OE9A107410, OE9A113003, and the GH18 class V OE9A070518 were also up–regulated by *V. dahliae* infection (**Figure 7A**).

From the ‘Galega vulgar’ experimental trial, the plants’ infection with *C. nymphaeae* was inexistent before fungi inoculation and its presence, was confirmed 30 days after inoculation (**Supplementary Figure 2**). Gene expression analysis revealed a general up–regulation of all the 6 analyzed genes (**Figure 7B**). However, significant differences were only found for genes OE9A087887 and OE9A025476 (GH19 class II), OE9A109397 (GH19 class IV) and OE9A010255 (GH18 class III) at 30 days post–infection (*P* < 0.05).

## 4 Discussion

Olive tree is affected by multiple fungal diseases (Moral et al., 2017; Fernández-Escobar, 2019; Markakis et al., 2021), for which modern cultivation systems such as high–density orchards may contribute to increase their incidence and severity. Chitinase enzymes play critical roles in plant defense against pathogenic fungal challenges (Grover, 2012; Pusztahelyi, 2018). In the present study, –28 putative chitinase and 5 chitinase–like genes were identified in the genome of *O. europaea* subsp. *europaea*, based on the “Farga” genome assembly OLEA9. Phylogenetic and domain analyses grouped OeChi into two main glycoside hydrolase families, GH18 and GH19, further subdivided into five classes (I–V), consistent with the established classification of plant chitinases (Passarinho and de Vries, 2002; Cao and Tan, 2019; Haxim et al., 2022; Zhang et al., 2022; He et al., 2023). The clear separation between GH18 and GH19 members and the clustering of chitinase-like proteins within the GH18 lineage suggest evolutionary conservation with potential functional diversification.

Gene duplication is a prevalent phenomenon and a major driving force in plant genome evolution. It takes place through various mechanisms, including tandem duplication, segmental duplication, and whole-genome duplication (Flagel and Wendel, 2009). Olive tree exhibits a distinct evolutionary history compared to other members of the Oleaceae family. In fact, phylogenomic analysis reveals that Olea has undergone specific polyploidization events, including a recent, olive-specific duplication event, as well as ancient allopolyploidization events. These events have contributed to its unique genomic makeup, making the olive tree quite divergent from other the members of the Lamiales order and even other members from the Oleaceae family (Julca et al., 2018). Consistently, our synteny analysis with *S. indicum* revealed a low level of collinearity. However, because not all *OeChi* genes were anchored to chromosomes, a comprehensive analysis could not be fully achieved.

In the present study, one segmental duplication was identified between chromosomes 6 and 11, along with three tandem duplication events on chromosomes 7, 9, and 12, all of which belong to the GH18 family. As duplicated genes often undergo functional diversification (Prince and Pickett, 2002; Panchy et al., 2016), the two segmentally duplicated *OeChi* genes may have experienced subfunctionalization, particularly since one displayed clear expression across tissues while the other was not detectably expressed. Nevertheless, our K*_a_*/K*_s_* analysis revealed that three chitinase gene pairs in olive are evolving under strong purifying selection. This pattern is consistent with observations in other plant species, including *P. trichocarpa* (Zhang et al., 2022), apple (Haxim et al., 2022), maize (Wang et al., 2024), lodgepole pine (*Pinus contorta* var. *latifolia* Engelm.) and jack pine (*Pinus banksiana* Lamb) (Peery et al., 2021) as well as tomato (Cao and Tan, 2019), where chitinase genes are similarly constrained by negative selection. The prevalence of purifying selection across these duplicated gene pairs highlights the strong functional constraints acting on chitinase genes, likely reflecting the need to preserve conserved catalytic domains essential for antifungal defense, although further functional analyses are necessary to clarify potential divergence between duplicated copies.

Gene structural variability was observed amongst members of the same family (GH18 or GH19). Similar structural variability within a gene family has previously been reported in the olive tree (Cardoso et al., 2015; Cardoso et al., 2022). In fact, intron gain and loss events have been recognized as key contributors to the evolutionary dynamics of intron architecture (Knowles and McLysaght, 2006). Intron sequence variability has been associated with functional role in gene expression regulation, largely due to their capacity to harbor regulatory elements, and they participate in gene expression and regulation by interacting with corresponding mRNA sequences (Zhang et al., 2016). TEs, known as self–replicating DNA segments that can insert themselves throughout the genome, play a substantial role in shaping genome architecture and modulating gene function through a variety of mechanisms (for review see Hirsch et al., 2006 (Hirsch and Springer, 2017)). Previous studies developed in olive tree gene families have enabled the identification of TEs within gene sequences, as well as in upstream and downstream flanking regions (Velada et al., 2018; Cardoso et al., 2022). In this study we identified a *Copia*–LTR retrotransposon within intron 1 of the OE9A061598 gene. TEs are widely recognized for their potential to confer adaptive advantages under environmental stress conditions (Negi et al., 2016). However, they may also preferentially insert into genes with low expression levels (Maumus and Quesneville, 2014), highlighting the importance of further investigating their role in gene regulation under stress conditions.

In accordance with previous reports, the transcription patterns of *O. europaea* chitinase genes from cv. ‘Picual’ RNA–seq data showed both constitutive and induced expression upon fungal infection (Xu et al., 2016; Bartholomew et al., 2019) (**Figures 6**, **7A**). Constitutively expressed chitinases reportedly participate in physiological functions such as flower and seed development, symbiotic interactions, cell division and programmed cell death (Cletus et al., 2013). Furthermore, chitinases present constitutively in storage tissues such as seeds, fruits and tubers might contribute as source of amino acids (Peumans et al., 2002). A signal peptide located at the N–terminus of most plant chitinases is responsible for their secretion after posttranslational modification (Taira et al., 2009). In this study, around 75% of the chitinase proteins were predicted to have signal peptides (**Table 1**), suggesting translocation from the cytosol into specific organelles. Furthermore, the presence of signal peptides in all *V. dahliae –* strongly induced *OeChi* genes suggests their secretion to the apoplast, where they may directly degrade fungal cell walls. This supports their role in extracellular antifungal defense. In apple, chitinase genes induced in response to *Valsa mali* infection all carried the signal peptide sequence, being suggested its secretion into the apoplast and involvement in plant–pathogen interaction (Haxim et al., 2022). Similarly to previous studies (Tobias et al., 2017; Haxim et al., 2022), we found that *OeChi* Class II genes greatly responded to fungal infection, as well as some GH18 Class III genes. Reinforcing their potential against fungal infection, it was for example reported that the overexpression of a barley Class II chitinase gene in transgenic wheat enhanced resistance to *Fusarium graminearum* in greenhouse and field conditions (Shin et al., 2008). However, we also observed that some genes from GH18 Class III and most from GH18 Class V consistently displayed low transcript accumulation. This is similar to what has also been observed in species like cotton or apple (Maumus and Quesneville, 2014; Haxim et al., 2022). Expression divergence is widely regarded as an early step in functional divergence following gene duplication, often preceding detectable structural or biochemical changes (Conant and Wolfe, 2008). Therefore, the reduced transcript abundance observed for some olive chitinases may reflect regulatory divergence and sub functionalization within the gene family.

In the experimental trial using cv. ‘Galega vulgar’ challenged with *C. nymphaeae* there was a consistent up–regulation of all the studies genes at 30 days post–infection (**Figure 6B**), although not significant for OE9A106107 (GH19 class I) and OE9A015656 (GH18 hevamine class III). It was also observed that the gene OE9A109397 (GH19 class IV), which was almost not detected in the RNA–seq data for *V. dahliae* infection, was clearly detected by RT–qPCR in ‘Galega vulgar’ infected with *C. nymphaeae*. Besides methodology specificities, it is possible that the interaction with different fungi leads to a differential expression of chitinase genes, as it has been observed in other plant species (Salzer et al., 2000; Peery et al., 2021).

Most of the sequences where signal peptides were not detected also showed little to no detectable expression, except for SI–CLP (OE9A118797) and GH18 class III (OE9A116561) genes, both of which presented a constitutive expression in nearly every tissue/organ (**Figure 6**, **7A**). In cucumber, SI–CLP was also reported to be constitutively expressed in several tissues, and similarly to olive tree SI–CLP, presents a large size and numerous exons (Bartholomew et al., 2019). SI–CLPs contain a glycosyl hydrolase family 18 domain but lack a chitin–binding domain, thus still being of unknown function (Rosa et al., 2016). Similarly, narbonins’ function is unclear. Narbonin is a globulin protein firstly identified in the eudicot *Vicia narbonensis* that lacks conserved chitinase catalytic residues and enzymatic activity (Tyler et al., 2010). The presence of signal peptides in narbonin CPL sequences is not always observed (Bartholomew et al., 2019; Cazares-Álvarez et al., 2024), and in this study two of *O. europaea* GH18 narbonins were not predicted to contain signal peptides and were consistently expressed at very low levels. Despite lacking enzyme function, CLPs have diversified to play key roles in plant defense, stress responses, and development. For instance, Gu et al. (Gu et al., 2019) demonstrated that CPL1 helps to regulate ethylene production and root development in Arabidopsis.

To explore the mechanism by which the chitinases are transcriptionally regulated, we analyzed the promoter regions of the 33 chitinase and chitinase–like genes. There were found numerous *cis*–elements involved in response to multiple stresses and hormone–related (**Figure 4**). We also detected a few pathogen–responsive *cis*–acting elements, such as the W–box, ERE and TGACG–motif. While some were observed in genes induced by fungal infection, others were also found in non–responsive genes. The important W–box has (C/TTGACT/C) as a core sequence and acts as a binding site for WRKY transcription factors (Eulgem et al., 2000). TGAC–containing W–box has been shown to mediate the fungal elicitor–induced gene expression by interacting with WRKY1 (Eulgem et al., 1999). The greater occurrence of the W–box *cis*–element in the GH18 Class III OE9A107410 and in the GH19 class II OE9A068866 gene might in fact be involved with their high responsiveness to fungal infection (**Figure 7A**). However, many *cis–*elements are involved in responses to both biotic and abiotic stresses, thus overlapping signaling pathways (Sheshadri et al., 2016), which might prove difficult to assure their involvement in specific stresses. Furthermore, genes that currently do not respond to biotic stress might have evolved from ancestral genes that were involved in biotic stress responses (Panchy et al., 2016). In Arabidopsis, duplicated gene pairs were shown to vary greatly in their *cis*–regulatory element architecture, resulting in changes in regulatory network connectivity (Arsovski et al., 2015). Also the persistence of *cis*–elements without a functional role might have neutral effects on the organism, meaning there is no evolutionary pressure to eliminate them. Therefore, a functional analysis is required to confirm *O. europaea* chitinases’ *cis*–elements in response to specific stress conditions.

## 5 Conclusions

Plant chitinases exhibit high diversity and play key roles in growth, development, and responses to biotic and abiotic stress. Identifying and characterizing these genes and proteins are essential steps for their application in biotechnology. This study provides the first comprehensive characterization of chitinase and chitinase-like genes in the *Olea europaea* genome. Expression profiling revealed substantial variation in the response of genes to fungal infection, with certain members showing a strong induction. Therefore, screening chitinase enzymes with high antifungal activity is important to meet the need to improve resistance against phytopathogens either by gene manipulation, the use of endophytes or recombinant proteins. Overall, our findings provide a valuable foundation for future strategies aimed at improving fungal disease resistance in olive tree.

## Data Availability

The datasets presented in this study can be found in online repositories. The names of the repository/repositories and accession number(s) can be found in the article/**Supplementary Material**.
