# Supplementary material for: Chitinase genes in olive tree (Olea europaea L.): evolutionary dynamics and stress–responsive regulation
Source: Front Plant Sci. 2026 Apr 17;17:1771535. doi: 10.3389/fpls.2026.1771535 (PMC13132863; doi:10.3389/fpls.2026.1771535)
Supplement: Supplementary file 2 [file DataSheet1.pdf]

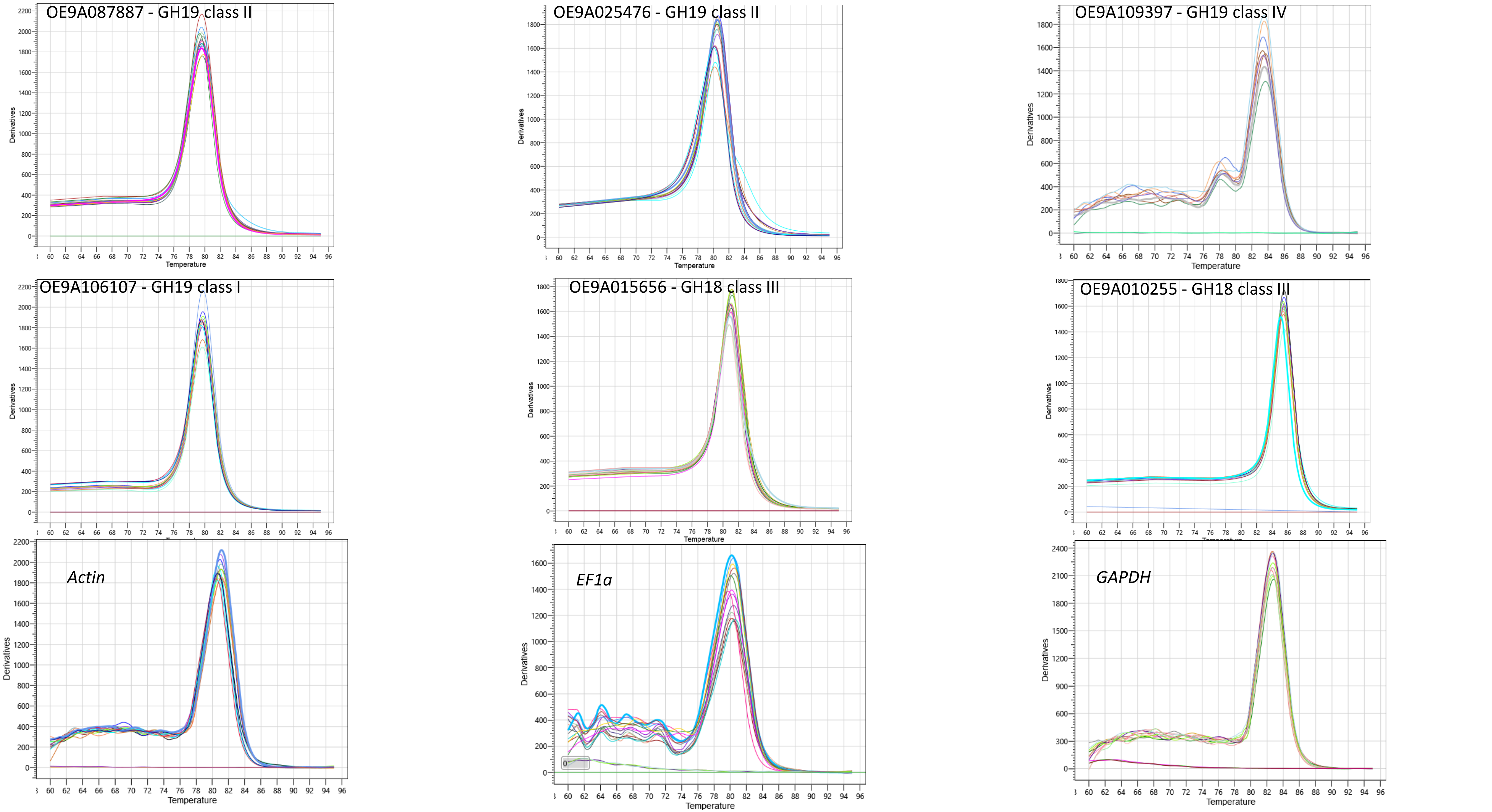

Figure S1. Primer specificity test through dissociation curve analysis. The reference genes were *Actin* (*ACT*), *Glyceraldehyde-3-phosphate dehydrogenase* (*GAPDH*) and *Elongation factor* (*EF1a*). The target chitinase genes belong to different classes from the GH18 and GH19 families.

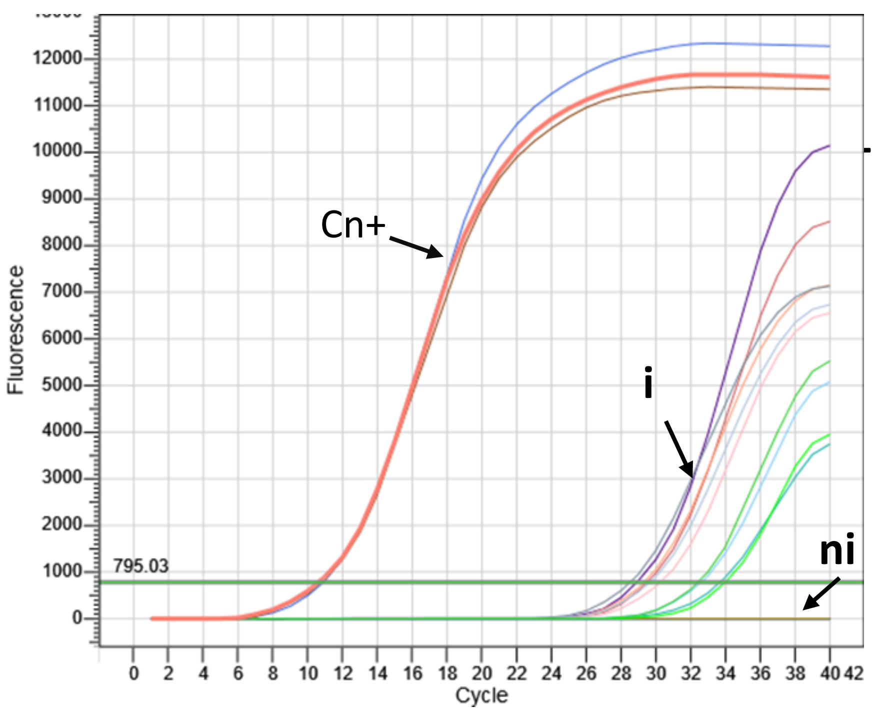

Figure S2: Amplification plot of *Colletotrichum nymphaeae* positive control (Cn+), olive leaves before fungi inoculation (ni), and olive leaves 30 days after inoculation with *C. nymphaeae* (i).

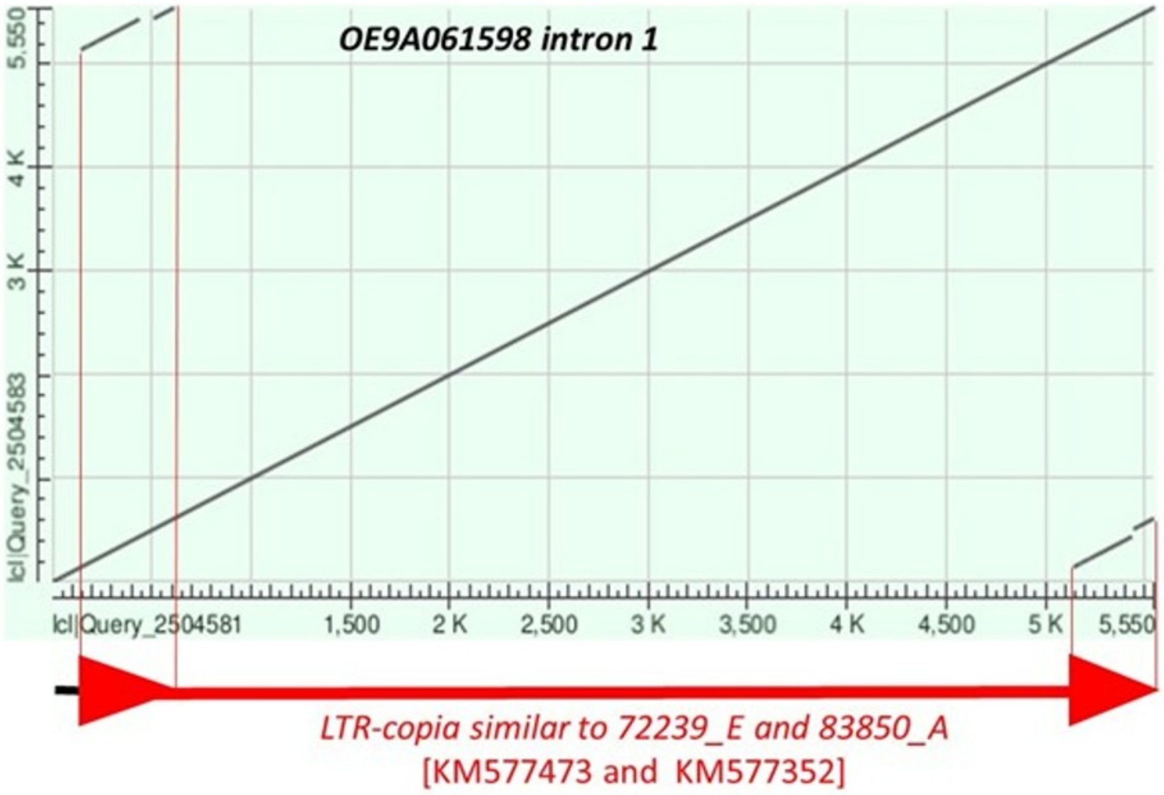

Figure S3. Schematic representation of the organization of intron 1 of the OE9A061598 gene in *Olea europaea* subsp. *europaea.* The red line represents the insertion of the *Copia*-LTR retrotransposon found similar to 72239_E (KM577473) and 83850_A (KM577352), and of *Copia*-74 MN from *Morus notabilis*, and red arrows show localization of its LTRs.
